# Research state of the herbal medicine Huangqi (Radix Astragali): A global and bibliometric study

**DOI:** 10.1097/MD.0000000000037277

**Published:** 2024-02-23

**Authors:** Yan-Jun Chen, Ming-Rong Xie, Sheng-Qiang Zhou, Fang Liu

**Affiliations:** a Hunan University of Chinese Medicine, Changsha, China; b National TCM Master Liu Zuyi Inheritance Studio, The Affiliated Hospital of Hunan Academy of Chinese Medicine, Changsha, China; c Nanjing University of Chinese Medicine, Nanjing, China.

**Keywords:** bibliometrics, CiteSpace, Huangqi (Radix Astragali), VOSviewer, Web of Science

## Abstract

**Background:**

Huangqi (Radix Astragali) is a natural medicine with a wide range of uses. The research related to Huangqi is getting hotter and the number of publications is gradually increasing. This study aims to explore the current status and emerging trends of Huangqi-related research.

**Method:**

Huangqi-related literature was systemically obtained from the Web of Science database. The CiteSpace, VOSviewer, and, R package “Bibliometrix” tools were used to analyze the number of publications, countries, research institutions, journals, authors, keywords, references, and trends.

**Results:**

A total of 2255 papers were retrieved for analysis. These papers were written by 11,247 authors from 1927 institutions in 71 countries, published in 570 journals, and cited 73,534 references from 11,553 journals. From 1999 to 2022, the number of publications gradually increased. China was the country with the highest number of publications. The most prolific institution was Shanghai University of Chinese Medicine. *Evidence-Based Complementary and Alternative Medicine* was the journal publishing the most Huangqi-related literature. Dr Karl Wah Keung Tsim was the authors with the most output publications. The Review, entitle “*Review of the Botanical Characteristics, Phytochemistry, and Pharmacology of Astragalus membranaceous (Huangqi),” was the reference* being cited most frequently. The major keywords were apoptosis, oxidative stress, and inflammation. Gut microbiota and epithelial-mesenchymal transitions were new research hotspots in recent years.

**Conclusion:**

This study used quantitative and visual analysis of Huangqi to provide insights into the research priorities, frontier research hotspots, and future research trends in this field.

## 1. Introduction

Huangqi, also known as Radix Astragali, Astragalus, and milkvetch, is a leguminous herb with wide distribution in temperate regions of Asia, North America, and Europe.^[1]^ The main components of Huangqi contain saponins, flavonoids, and polysaccharides.^[2]^ Modern pharmacological studies indicated that Huangqi shows a positive effect in anti-oxidant, anti-inflammatory, anti-apoptotic, anti-aging, hypotensive, and immunomodulatory effects.^[3,4]^ According to traditional Chinese medicine (TCM) theory, Huangqi was sweet, slightly warm, belonging to the lung and spleen meridians, with important roles in raising the “yang qi” in the surface, promoting water drainage, and decreasing swelling. Huangqi was one of the core ingredients in many traditional Chinese medicine prescriptions, such as Huangqi Jianzhong Tang for the treatment of chronic gastritis^[5]^ and Huangqi Guizhi Wuwu Tang for diabetic peripheral neuropathy.^[6]^

Since the 20th century, Huangqi-related research has gradually become one of the hotspots in TCM research, with increasing publications. However, to our knowledge, there is no study investigating Huangqi-related research from a bibliometric and quantitative perspective.

Bibliometric analysis, a statistical methodology, utilizes visual representations to forecast knowledge structure and identify research hotspots within a specific field of study.^[7]^ In this study, we conducted the first bibliometric study in the research field of Huangqi-related research, exploring the hotspots and frontiers and generating the corresponding knowledge maps. The presented study provided insights into the latest progress, evolution paths, frontier research hotspots, and future research trends in the field.

## 2. Methods

### 2.1. Search strategy and data acquisition

The Web of Science (WoS) database is a bibliographic resource containing more than 20,000 academic publications covering 250 different research fields worldwide,^[8]^ which is believed to be the most suitable database for bibliometric analyses.^[9]^

The literature analyzed in this study was obtained from the WoS database (SCI-EXPANDED & SSCI). The search formula was “TS = (radix astragali) OR (astragalus) OR (Huangqi) OR (milkvetch)” and the time was set before January 1, 2023. Literature that did not match the study and duplicates were manually excluded.

### 2.2. Data analysis

Data were visualized and analyzed by using CiteSpace, VOSviewer, and R package “Bibliometrix” software.

CiteSpace, a software developed by Dr Chao-Mei Chen in 2004,^[10]^ was used to generate visual knowledge maps and clearly show the keywords, hotspots, and development trends in the research fields.^[11]^ It has become a key tool for bibliometric analysis in recent years.^[12]^

The VOSviewer was created by Leiden University Center for Science and Technology Studies in 2010.^[13]^ As a software for creating and analyzing bibliometric networks, VOSviewer integrates literature information and visualizes authors, institutions, and keywords. R package “Bibliometrix” (https://www.bibliometrix.org) was launched in 2017 by Dr Massimo Aria and Dr Corrado Cuccurullo, which is a comprehensive mapping analysis tool for bibliometric analysis.^[14]^

## 3. Results

### 3.1. The scientific outcomes of Huangqi-related research

**Figure 1.**
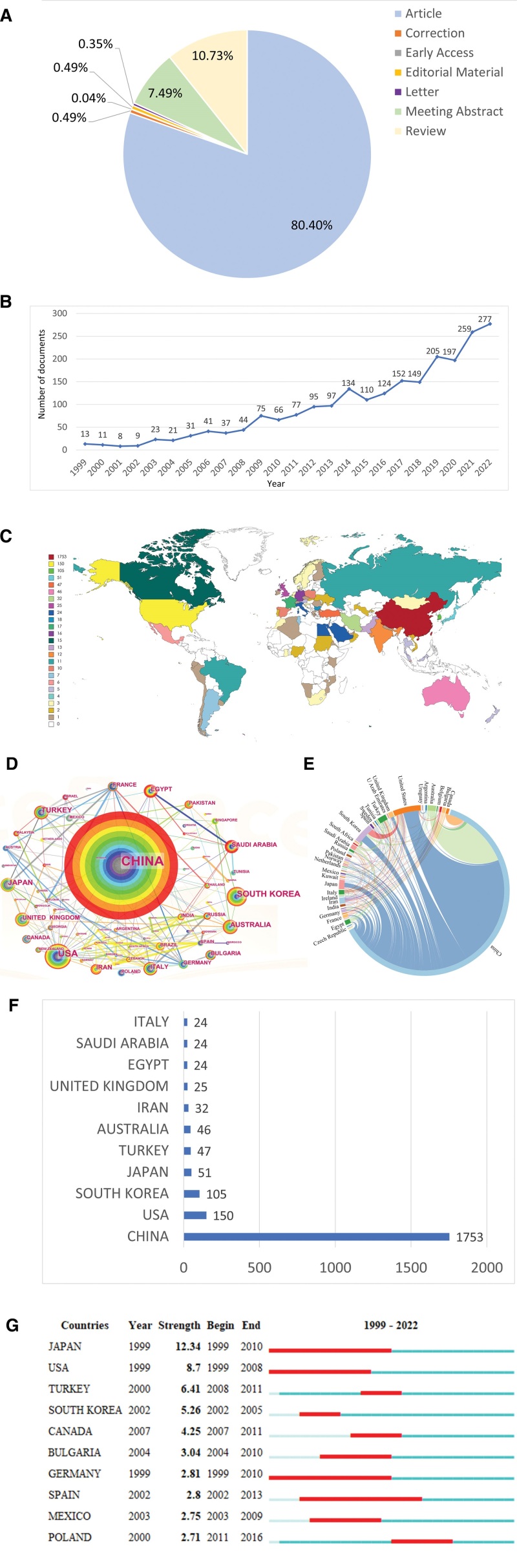
Study results and analysis of countries. (A) Type and proportion of literature included in the analysis. (B) Trend of the number of Huangqi-related papers from 1999 to 2022. (C) Geographical distribution of countries worldwide. (D) Cooperation between countries. The circle represents the country. The larger the circle, the greater the number of papers. The thicker the line between the circles, the closer the cooperation. (E) Distribution of countries in the field of Huangqi. The size of the circle represented the number of papers published by countries, and the line represented cooperation among countries. (F) The top 10 countries in terms of number of papers. (G) Top 10 countries with the strongest outbreak. The red bars represented burst duration.

A total of 2255 papers were taken into analysis in this study. The literature types were mainly article and review, including 1813 articles (80.4%), 242 reviews (10.73%), 169 meeting abstracts (7.49%), 11 corrections (0.49%), 11 editorial material (0.49%), 8 letters (0.35%), and 1 early access (0.04%) (Fig. 1A). These papers were written by 11,247 authors from 1927 institutions in 71 countries, published in 570 journals, and cited 73,534 references from 11,553 journals.

### 3.2. Temporal trend of publication outputs

The number of publications in each period reflected the research trend. A total of 2255 publications on Huangqi were published from 1999 to 2022, with the number of publications increasing year by year (Fig. 1B). Overall, it can be divided into 2 phases. The first phase, from 1999 to 2013, was in the slow start period. In the second stage, from 2014 to 2022, the number of publications showed a rapidly increasing trend. The year 2022 had the most publications in recent years, which peaked at 277. It is expected that publications may continue to increase in the future.

### 3.3. National geographic distribution and collaboration of publications

According to the geographic distribution heat map (Fig. 1C), Asia and North America had the highest number of papers. Huangqi-related research was conducted in 71 countries, and showed international cooperation (Fig. 1D). China, the United States, Japan, and South Korea cooperated closest (Fig. 1E). Among the top 10 countries (Fig. 1F), China published the most literature (1753 publications, 77.74%), which was more than 10 times higher than other countries, followed by the United States (150 publications, 6.65%) and South Korea (105 publications, 4.66%).

Burst detection identifies abrupt changes in information through analysis of intensity and duration. The outbreak countries of Huangqi-related research were booming spanned 1999 to 2016 but disappeared after 2016 (Fig. 1G). Six countries, including Japan, the USA, Turkey, South Korea, Canada, and Bulgaria, had significantly stronger outbreaks. Japan showed the strongest strength from 1999 to 2010, conducting a lot of research on Huangqi, with an outbreak intensity of 12.34.

### 3.4. Research institutions

**Figure 2.**
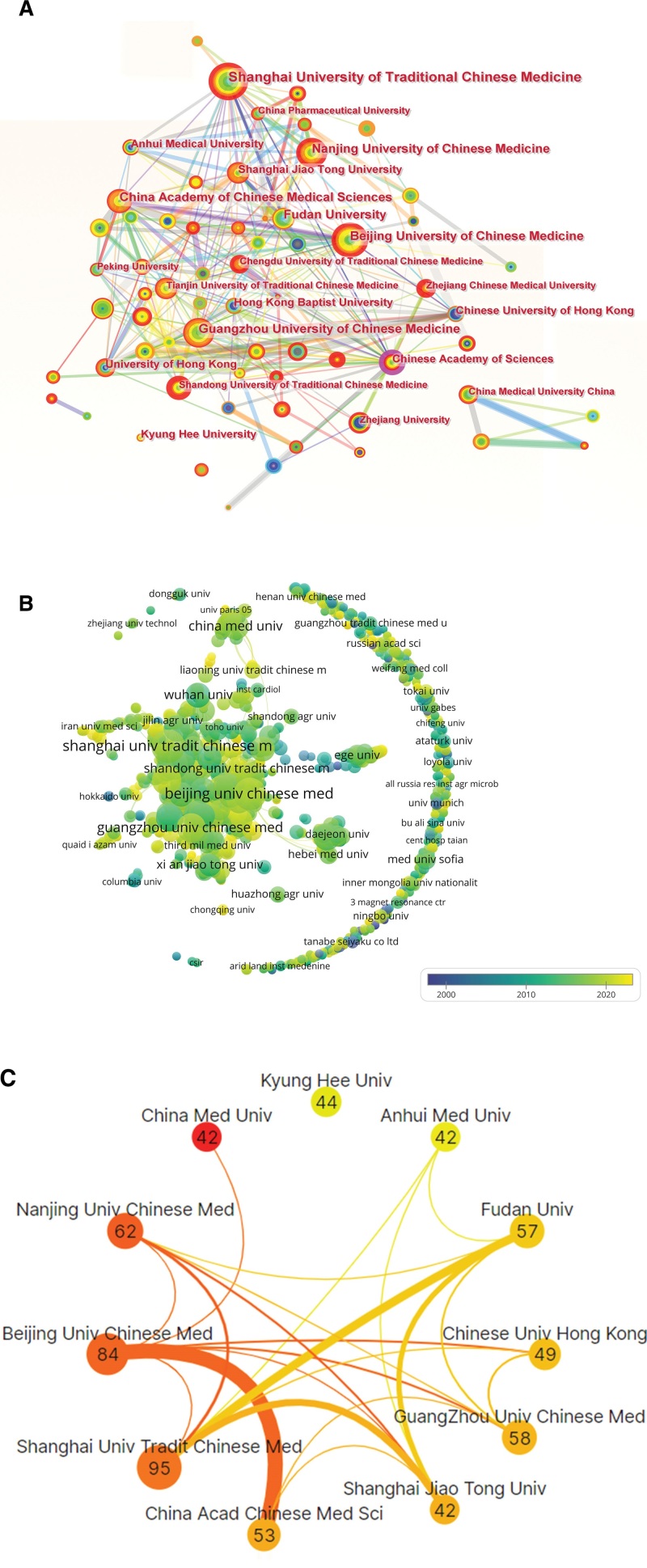
Analysis of institutions. (A)The circle represented the institution. The larger the circle, the greater the number of papers. The thicker the line between the circles, the closer the cooperation. (B) Time line visualization of research institution. The circle represented the research institution. The larger the circle, the more publications, the lighter the color, and the later the publication year. (C) Cooperation among the top 10 study institutions. The number in the circle showed the number of papers. The darker the circles and the thicker the connecting lines, the closer the cooperation.

The symbiosis diagram showed that most of the research institutions were from China (Fig. 2A). The majority of research institutions entered this field and published literature after 2010 (Fig. 2B), which was consistent with the publication trend (Fig. 1B). Among the top 10 institutions (Table S1, Supplemental Digital Content, http://links.lww.com/MD/L717), 9 institutions were from China, except for Kyung Hee University from South Korea. Unsurprisingly, 5 of the top 10 institutions were universities of TCM. Shanghai University of Chinese Medicine published the most literature (95 literature, 4.21%), followed by Beijing University of Chinese Medicine (84 literature, 3.72%), and Nanjing University of Chinese Medicine (62 literature, 2.75%). Some institutions work closely with each other, such as Beijing University of Chinese Medicine, China Academy of Chinese Medical Sciences, and Shanghai University of Chinese Medicine. Kyung Hee University conducted Huangqi-related research independently, rather than in collaboration with other institutions (Fig. 2C).

### 3.5. Journals and co-cited journals

**Figure 3.**
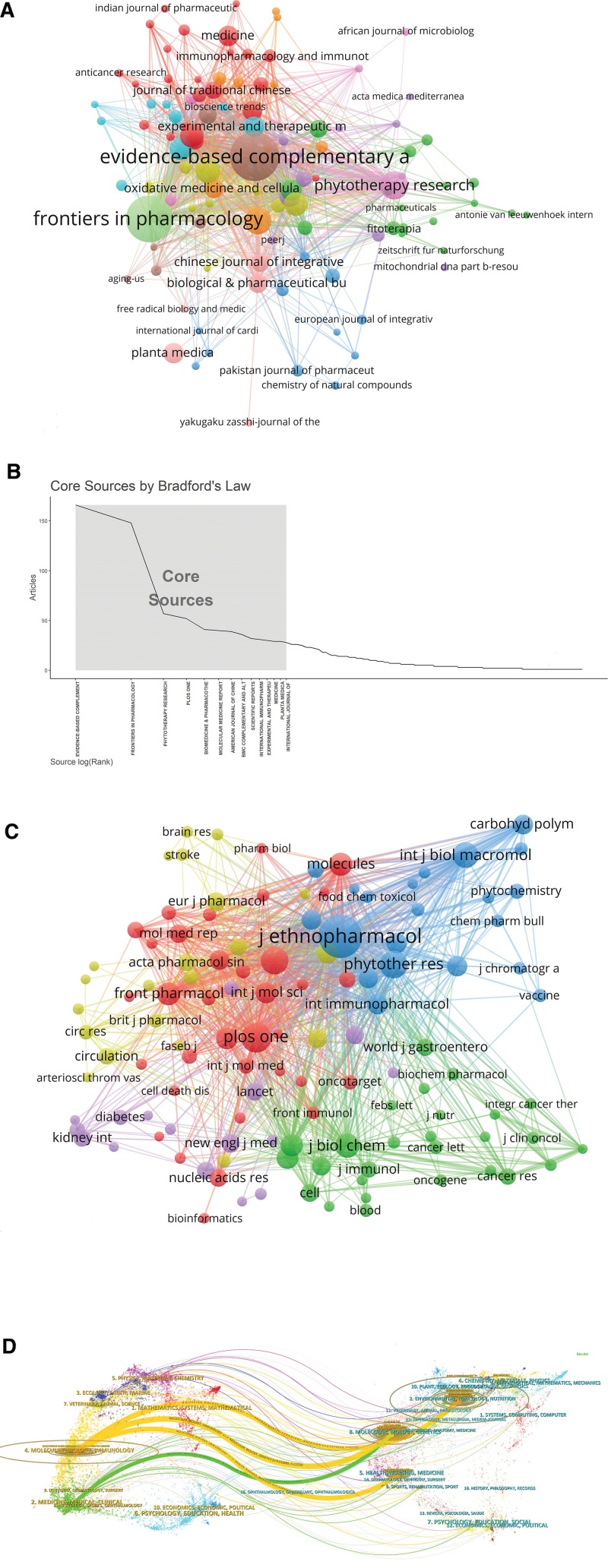
Analysis of journals and co-cited academic journals. (A) The circle represented the journals. The larger the circle, the greater the number of papers. (B) Core journals. (C) The circle represented the co-cited journals. The larger the circle, the greater the number of papers. (D) The dual-map overlay of journal. The thicker the line, the more citation. Citing journals on the left and cited journals on the right, and the curve is the citation line.

Huangqi-related literature was included in 570 journals (Fig. 3A). According to Bradford’s Law,^[15]^ 14 core journals were identified (Fig. 3B; Table S2, Supplemental Digital Content, http://links.lww.com/MD/L718). The majority of core journals were related to pharmacology research and/or integrative medicine, publishing 758 papers that accounted for 33.61% of the total number of literature, with impact factors ranging from 1.817 to 7.419. Among the core journals, the top with the most publications were *Evidence-Based Complementary and Alternative Medicine* (166 publications, 7.36%). The journal with the highest impact factor was *Biomedicine and Pharmacotherapy* (Impact Factor = 7.419). The journal, *International Immunopharmacology*, published only 31 papers, but with the highest average number of citations per article (43.9 times/publication), indicating that this journal also had a strong influence on the research field of Huangqi.

The co-cited journal visual analysis was shown in Figure 3C. Among the top 10 co-cited journals, 6 journals belonged to Journal Citation Reports Q1 (Table S3, Supplemental Digital Content, http://links.lww.com/MD/L719). The impact of journals depends on the number being co-cited. The most co-cited journals were *Journal of Ethnopharmacology* (2408 co-citations), indicating that it had a significant impact in this field, followed by *Plos One* (1141 co-citations) and *Phytotherapy Research* (959 citations).

A dual-map overlay graph of journals was overlaid to indicate links between journals. There were 4 main citation paths in this research area, including 3 in orange and 1 in green (Fig. 3D). The orange path indicated that literature published in journals of molecular/biology/immunology frequently cited literature published in journals of health/nursing/medicine, molecular/biology/genetics, and nutrition/toxicology/environment. The green path indicated that literature published in medicine/medical/clinical often cited literature published in journals of molecular/biology/genetics.

### 3.6. Author and author bursts

**Figure 4.**
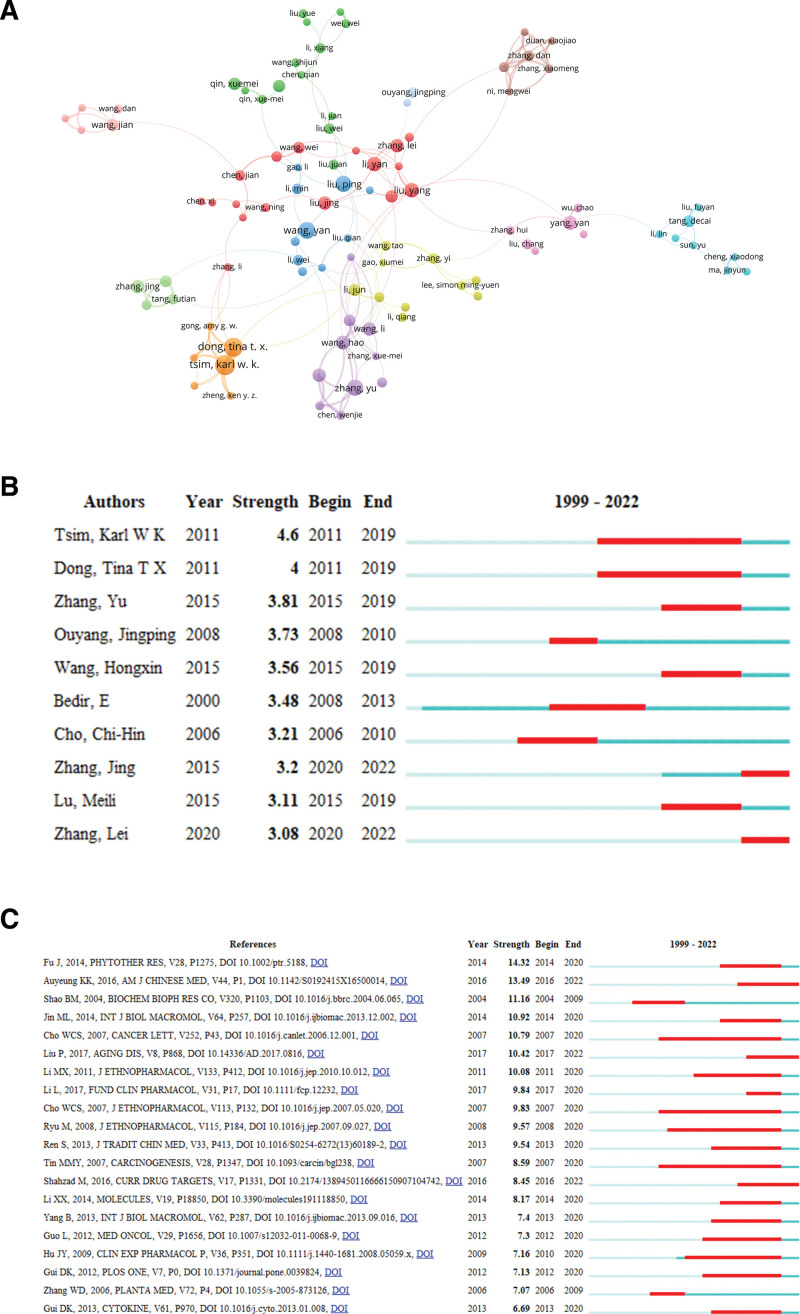
Analysis of authors and references. (A) Major core authors. The circle represents the author. The larger the circle, the greater the number of papers. The thicker the line between the circles, the closer the cooperation. (B) Top 10 outbreak authors. The red bars represented burst duration. (C) Top 20 references with the strongest citation bursts.

The authors were analyzed to get the core scholars and the main research power in the current research field. Huangqi-related research was conducted by 11247 authors. Among all authors, the author with the most publications were Dr Karl Wah Keung Tsim (21 papers), followed by Dr Tina Ting-Xia Dong (19 papers), and Dr Yan Wang (16 papers) (Table S4, Supplemental Digital Content, http://links.lww.com/MD/L720). Dr Ping Liu was the most influential author with the highest average number of citations per literature (35.13 citations/literature). Figure 4A shows that there are primarily 2 research teams involved in author collaboration, led by Dr Karl Wah Keung Tsim. and Dr Tina Ting-Xia Dong, who frequently and closely collaborate with each other. Dr Karl Wah Keung Tsim (Strength = 4.6) and Dr Tina Ting-Xia Dong (Strength = 4) were also the most outbreak authors with the longest duration of outbreak years (2011–2019) (Fig. 4B).

### 3.7. References and co-cited references

The top 10 references were listed in Table S5, Supplemental Digital Content, http://links.lww.com/MD/L721 which were published between 2014 and 2020 were mainly pharmacological reviews. The review, “*Astragalus membranaceus: A Review of its Protection Against Inflammation and Gastrointestinal Cancers*,*”*^[16]^ was cited mostly (54 times). “*Anti-Aging Implications of Astragalus Membranaceus (Huangqi): A Well-Known Chinese Tonic*”^[17]^ and “*The Antioxidant Effects of Radix Astragali (Astragalus membranaceus and Related Species) in Protecting Tissues from Injury and Disease*”^[3]^ were new outbreak cited references emerging in the last 5 to 6 years.

If 2 or more documents are cited by one or more subsequent documents at the same time, the 2 documents constitute a co-citation relationship, which can be used as a measure of the degree of relationship between articles. Table S6, Supplemental Digital Content, http://links.lww.com/MD/L722 showed the top 10 most frequently co-cited references. The literature, “*Review of the Botanical Characteristics, Phytochemistry, and Pharmacology of Astragalus membranaceus (Huangqi*)”^[18]^ was also co-cited mostly (109 times). Unexpectedly this paper also had the strongest citation burst (Strength = 14.32) (Fig. 4C).

### 3.8. Keywords

**Figure 5.**
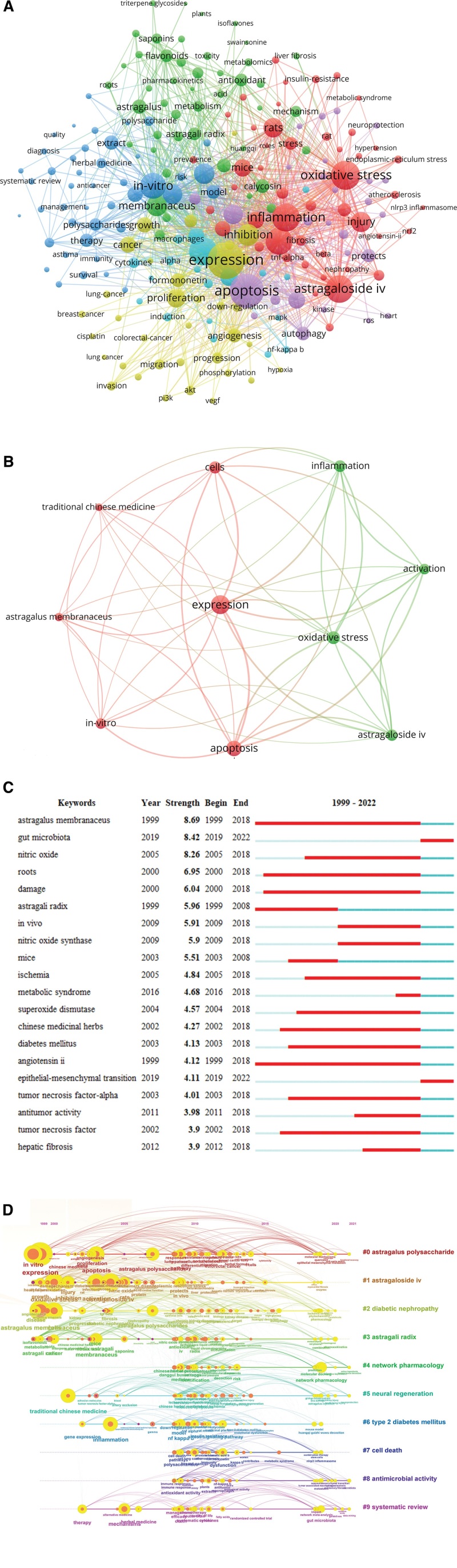
Analysis of keywords. (A) Main keywords. The circle represented the keyword. The larger the circle, the greater the number of papers. The thicker the line between the circles, the closer the cooperation. (B) Top 10 keywords. The larger the circle, the higher the frequency of keywords. (C) Top 20 outbreak keywords. The red bars represented burst duration. (D) Keyword timeline chart. The circle represented the keyword. The larger the circle, the greater the number of papers. The thicker the line between the circles, the closer the cooperation.

By analyzing the main keywords (Fig. 5A), we can summarize the hotspots and directions in the research field. The top 10 keywords were mainly related to pharmacological mechanisms (Fig. 5B; Table S7, Supplemental Digital Content, http://links.lww.com/MD/L723), including expression (322 times), apoptosis (261 times), oxidative stress (217 times), cells (203 times), inflammation (198 times), astrogaloside iv (194 times), in vitro (181 times), activation (179 times), astrogalus membranaceus (141 times), traditional Chinese medicine (133 times).

The burst of keywords reveals the hotspots of research and higher attention in a certain period. The red line indicated the duration of the appearance of the outbreak words (Fig. 5C), and the top 3 outbreak words were “astragalus membranacens” (Strength = 8.69), “gut microbiota” (Strength = 8.42), and “nitric oxide” (Strength = 8.26). The top 3 in duration were “astragalus membranacens” (1999–2018, 19 years), “angiotensin ii” (1999–2018, 19 years), “roots” (2000–2018, 19 years), and “damage” (2000–2018, 18 years). Among the top 20 outbreak keywords, 18 keywords outbreak spanned from 1999 to 2018. Since 2019, “gut microbiota” and “epithelial-mesenchymal transition” became new outbreak keywords.

The keyword timeline diagram indicated the stage characteristics and evolution of the current research field. Figure 5D showed the stage hotspots and research directions of Huangqi-related research from a temporal perspective. The research hotspots of Huangqi were changing. From 1999 to 2010, most of the studies were related to molecular mechanisms, including in vivo and in vitro studies, while from 2010 to 2022, most of the studies were focused on therapeutic, network pharmacology, and signaling pathways.

## 4. Discussion

In recent years, the field of Huangqi-related research has received increasing attention, with a growing number of publications. In this study, we applied bibliometric research methods to quantitatively analyze the scientific literature in the field of Huangqi, including inter-country collaboration, research institutions, active journals, core authors, keywords, and references, which may help to identify the latest progress, evolutionary pathways, frontier research hotspots and future research trends in this field.

Since 1999, the number of Huangqi-related literature was increasing, with rapid growth starting in 2009 and the peak annual published articles of 277 in 2022 (Fig. 1B), indicating that this field has a promising research future. These outcomes showed that studies on Huangqi had gained increasing attention from researchers from all over the world in recent years, and this research field was currently in a steady developmental stage.

Huangqi-related research is related to its geographical distribution mainly of Asia and North America.^[1]^ For countries, China was the leading research force in this field, and the United States and South Korea were also active countries according to the analysis of the collaboration network diagram (Fig. 1D). China was also the country with the most publications and cooperated closely with the United States, Japan, and South Korea. For institutions, 9 of the top 10 prolific institutions were from China, except for Kyung Hee University from South Korea. Unsurprisingly, 5 of the top 10 institutions were TCM universities, which collaborated closely with others. Strengthening the cooperation between TCM universities and other institutions may promote the development of this field.

Analysis of the literature sources distribution helped to find the core journals. *Evidence-Based Complementary and Alternative Medicine* was the journal that published the most literature, and researches related to Huangqi were widely welcomed in this journal (IF = 2.65, Q3). *International Immunopharmacology* was the most influential journal with the highest number of citations (IF = 7.149, Q1), which potentially reflected the close attention of Huangqi in immune-related research. The *Journal of Ethnopharmacology* was the journal being cited mostly, with an impact factor of 5.195. Furthermore, Huangqi-related research was mostly published in molecular/biology/immunology and medicine/medical/clinical journals, which may indicate that immunologic mechanism and clinical application research were the current focus.

Among the core authors of Huangqi, Dr Karl Wah Keung Tsim and Dr Tina Ting-Xia Dong were the top 2 most prolific authors, and also the first and second outbreak authors. The 2 scholars, both from Hong Kong University of Science and Technology, worked closely together to study the active pharmacological components of Huangqi,^[19]^ as well as the efficacy^[20]^ and mechanism of herbal formulas containing Huangqi,^[21]^ particularly Danggui Buxue Tang. The third most prolific Dr Yan Wang, from Wenzhou Medical University, focused on the research of Huangqi in heart diseases; he found that Huangqi exerted antiviral, anti-inflammatory, and anti-apoptotic effects in the treatment of viral myocarditis.^[22]^ In addition, Huangqi was found to promote angiogenesis, improve myocardial ischemia, and protect the heart.^[23]^ Dr Ping Liu, from Shanghai University of Chinese Medicine, was the most influential author. His research focused on the mechanism and role of Huangqi in liver diseases, revealing that Huangqi had the protective effect of anti-liver fibrosis^[24]^ and reduced cholestasis.^[25]^

It was noteworthy that apoptosis, oxidative stress, and inflammation appeared more frequently in the keywords, which may reflect the research focus of this field. Apoptosis: apoptosis is one of the core pathological features of many diseases. Huangqi may mediate apoptosis by reducing oxidative stress up-regulating anti-apoptotic protein Bcl-X and down-regulating pro-apoptotic Bax protein expression.^[26]^ Besides, Huangqi regulated the caspase-3-dependent pathway^[27]^ and down-regulated ATF2 and PERK factor expression in the endoplasmic reticulum stress pathway^[28]^ to inhibit apoptosis. Oxidative stress: sustained oxidative stress initiated the apoptotic program, and inhibition of oxidative stress decreased the damage of the body.^[29]^ Huangqi reduced oxidative stress by restraining ROS production,^[30]^ improving mitochondrial dysfunction,^[31]^ and enhancing Nrf2 activation and HO-1 and NQO1 expression.^[32]^ Inflammation: Huangqi mediated multiple signaling pathways to exert anti-inflammatory effects, such as mediating the TLR-4/NF-kb p65 pathway^[33]^ and regulating the Wnt/β-catenin signaling pathway,^[34]^ down-regulating the expression of inflammatory factors IL-1, IL-6, and TNF-α, and inhibiting the JNK signaling pathway associated with oxidative stress, reducing the expression of transcriptional level of the *puc* gene.^[35]^ These 3 pathological processes may be the main pharmacological mechanism of Huangqi, which was also the focus of the research area of Huangqi.

Keyword clustering showed that Huangqi-related studies focused on astragalus polysaccharide, astragaloside iv, diabetic nephropathy, and neural regeneration levels. These research hotspots were related but also distinct. Astragalus polysaccharide and astragaloside iv were the main active ingredients of Huangqi. Astragalus polysaccharide can modulate immunity, decrease blood lipids, regulate blood glucose, and antiviral, which was widely used in liver and kidney diseases.^[36]^ Astragaloside iv, with anti-inflammatory, anti-oxidant, and anti-apoptotic effects, was mainly used in heart, brain, and kidney diseases.^[37]^ Huangqi can reduce fasting blood glucose and urinary protein expression, improving glomerular hyperfiltration and protecting against diabetic nephropathy.^[38]^ It also enhanced antioxidant capacity and delayed kidney damage.^[39]^ The Chinese herbal formula Buyang Huanwu decoction, in which Huangqi is used as the sovereign medicinal, can rescue the nerves damaged by cerebral ischemia.^[40]^ Astragalus polysaccharide can also accelerate angiogenesis and promote axonal and myelin regeneration by activating the AKT/eNOS signaling pathway, presenting a positive effect on neural regeneration.^[41]^

The gut microbiota and epithelial-mesenchymal transitions were the outbreak keywords in the last 4 years, potentially indicating new hotspots in the field of Huangqi-related research (Fig. 5C). The gut microbiota plays a key role in a variety of diseases. It is suggested that Huangqi repaired the damaged intestinal barrier by regulating the intestinal microbiota,^[42]^ improving cognitive impairment^[43]^ and hypoglycemia,^[44]^ and lowering uric acid.^[45]^ The role of epithelial-mesenchymal transition was a major factor in the development of the epithelium. The epithelial-mesenchymal transition is one of the pathogenic mechanisms of many fibrotic diseases, and Huangqi can alleviate fibrosis by mediating multiple signaling pathways. For example, inhibition of AKT/GSK3β/β-catenin signaling pathway down-regulated epithelial-mesenchymal transition to reduce lung fibrosis.^[46]^ The TGF-β1/Smad signaling pathway was used to inhibit the epithelial-mesenchymal transition of renal tubules to improve renal interstitial fibrosis.^[47]^ New research hotspots may be the frontiers of future exploration, with great potential for exploration and reference value.

This study had some limitations. Firstly, the retrieved data were obtained from the WoS database and only English literature was included, which may leave out a small number of literature in other languages. Secondly, some important research, especially some newly published literature due to the possible time lag, may not attract enough attention from scholars at present.

## 5. Conclusion

In conclusion, this study conducted a bibliometric analysis of Huangqi-related research, identifying the important countries, journals, and authors, determining the research focus and hot frontiers, and revealing the current status and emerging trends in the Huangqi-related research field. Gut microbiota and epithelial-mesenchymal transitions mediated fibrosis emerged to be the hotspot of Huangqi-related research in the last 3 years, which may be the next direction for the future study of pharmacological research of Huangqi. Overall, this study may help investigators by providing an intuitive understanding of the research status of Huangqi.

## Acknowledgments

The help of related authors participating in this work is greatly appreciated.

## Author contributions

**Data curation:** Ming-Rong Xie.

**Writing – original draft:** Yan-Jun Chen.

**Writing – review & editing:** Fang Liu, Sheng-Qiang Zhou.

## Supplementary Material














