# Supplementary material for: Research state of the herbal medicine Huangqi (Radix Astragali): A global and bibliometric study
Source: Medicine (Baltimore). 2024 Feb 23;103(8):e37277. doi: 10.1097/MD.0000000000037277 (PMC11309597; doi:10.1097/MD.0000000000037277)
Supplement: Supplementary file 1 [file medi-103-e37277-s001.docx]

**Table S1. The top 10 productive institutions.**

| Rank | Country | Research institutions | Number of douvuments |
| --- | --- | --- | --- |
| 1 | China | Shanghai University of Traditional Chinese Medicine | 95 |
| 2 | China | Beijing University of Chinese Medicine | 84 |
| 3 | China | Nanjing University of Chinese Medicine | 62 |
| 4 | China | Guangzhou University of Chinese Medicine | 58 |
| 5 | China | Fudan University | 57 |
| 6 | China | China Academy of Chinese Medical Sciences | 53 |
| 7 | China | The Chinese University of Hong Kong | 49 |
| 8 | South Korea | Kyung Hee University | 44 |
| 9 | China | Shanghai Jiao Tong University | 42 |
| 9 | China | China Medical University | 42 |
| 9 | China | Anhui Medical University | 42 |
