# Supplementary material for: Research state of the herbal medicine Huangqi (Radix Astragali): A global and bibliometric study
Source: Medicine (Baltimore). 2024 Feb 23;103(8):e37277. doi: 10.1097/MD.0000000000037277 (PMC11309597; doi:10.1097/MD.0000000000037277)
Supplement: Supplementary file 2 [file medi-103-e37277-s002.docx]

**Table S2. List of the core journals.**

| Rank | Journal | Documents | Citations | IF | JCR |
| --- | --- | --- | --- | --- | --- |
| 1 | Evidence-Based Complementary and Alternative Medicine | 166 | 1445 | 2.65 | Q3 |
| 2 | Frontiers In Pharmacology | 148 | 1433 | 5.988 | Q1 |
| 3 | Phytotherapy Research | 57 | 1941 | 6.388 | Q1 |
| 4 | Plos One | 52 | 1802 | 3.752 | Q2 |
| 5 | Biomedicine & Pharmacotherapy | 41 | 717 | 7.419 | Q1 |
| 6 | Molecular Medicine Reports | 40 | 736 | 3.423 | Q3 |
| 7 | American Journal of Chinese Medicine | 39 | 1046 | 6.005 | Q1 |
| 8 | BMC Complementary and Alternative Medicine | 36 | 819 | 4.782 | Q1 |
| 9 | Scientific Reports | 32 | 825 | 4.996 | Q2 |
| 10 | International Immunopharmacology | 31 | 1361 | 5.714 | Q1 |
| 11 | Experimental And Therapeutic Medicine | 30 | 369 | 2.751 | Q4 |
| 12 | Medicine | 29 | 107 | 1.817 | Q3 |
| 13 | Planta Medica | 29 | 14 | 3.007 | Q2 |
| 14 | International Journal of Clinical and Experimental Medicine | 28 | 129 | 1.422 | Q4 |
