# Supplementary material for: Research state of the herbal medicine Huangqi (Radix Astragali): A global and bibliometric study
Source: Medicine (Baltimore). 2024 Feb 23;103(8):e37277. doi: 10.1097/MD.0000000000037277 (PMC11309597; doi:10.1097/MD.0000000000037277)
Supplement: Supplementary file 3 [file medi-103-e37277-s003.docx]

**Table S3. Top 10 cited journals.**

| Rank | Journal | Co-citated times | IF | JCR |
| --- | --- | --- | --- | --- |
| 1 | Journal of Ethnopharmacology | 2408 | 5.195 | Q1 |
| 2 | Plos One | 1141 | 3.752 | Q2 |
| 3 | Phytotherapy Research | 959 | 6.388 | Q1 |
| 4 | Evidence-Based Complementary and Alternative Medicine | 953 | 2.65 | Q3 |
| 5 | International Journal of Biological Macromolecules | 826 | 8.025 | Q1 |
| 6 | Journal of Biological Chemistry | 758 | 5.486 | Q2 |
| 7 | Frontiers In Pharmacology | 661 | 5.988 | Q1 |
| 8 | Molecules | 653 | 4.927 | Q2 |
| 9 | Nature | 648 | 69.504 | Q1 |
| 10 | Proceedings of the National Academy of Sciences of The United States of America | 601 | 12.779 | Q1 |
