# Supplementary material for: Research state of the herbal medicine Huangqi (Radix Astragali): A global and bibliometric study
Source: Medicine (Baltimore). 2024 Feb 23;103(8):e37277. doi: 10.1097/MD.0000000000037277 (PMC11309597; doi:10.1097/MD.0000000000037277)
Supplement: Supplementary file 4 [file medi-103-e37277-s004.docx]

**Table S4. Top 10 authors.**

| Rank | Author | Documents | Citations | Average number of citations | Country | Institution |
| --- | --- | --- | --- | --- | --- | --- |
| 1 | Karl Wah Keung Tsim | 21 | 353 | 16.81 | China | Hong Kong University of Science & Technology |
| 2 | Tina Ting-Xia Dong | 19 | 329 | 17.32 | China | Hong Kong University of Science & Technology |
| 3 | Yan Wang | 16 | 244 | 15.25 | China | Northwest A&F University - China |
| 4 | Yu Zhang. | 15 | 204 | 13.6 | China | School of Stomatology, Shanghai Jiao Tong University |
| 5 | Ping Liu | 15 | 527 | 35.13 | China | Shanghai University of Traditional Chinese Medicine |
| 6 | Yan Li | 13 | 192 | 14.77 | China | Xuanwu Hospital, Capital Medical University |
| 7 | Yang Liu | 13 | 152 | 11.69 | China | University of Chinese Academy of Sciences |
| 8 | Hao Wang | 12 | 192 | 16 | China | Shanghai University of Traditional Chinese Medicine |
| 8 | Ilina Krasteva | 12 | 77 | 6.42 | Bulgaria | Medical University Sofia |
| 8 | Seong-Gyu Ko | 12 | 169 | 14.08 | South Korea | Kyung Hee University |
| 8 | Yan Yang | 12 | 158 | 13.17 | China | Southwest Medical University |
