# Supplementary material for: Research state of the herbal medicine Huangqi (Radix Astragali): A global and bibliometric study
Source: Medicine (Baltimore). 2024 Feb 23;103(8):e37277. doi: 10.1097/MD.0000000000037277 (PMC11309597; doi:10.1097/MD.0000000000037277)
Supplement: Supplementary file 5 [file medi-103-e37277-s005.docx]

**Table S5. Top 10 references.**

| Rank | Title | Type | Citation times | Year | Journal | Reference |
| --- | --- | --- | --- | --- | --- | --- |
| 1 | Astragalus membranaceus: A Review of its Protection Against Inflammation and Gastrointestinal Cancers | Review | 54 | 2016 | The American Journal of Chinese Medicine | ^1^ |
| 2 | Anti-Aging Implications of Astragalus Membranaceus (Huangqi): A Well-Known Chinese Tonic | Review | 51 | 2017 | Aging and disease | ^2^ |
| 3 | Research review on the pharmacological effects of astragaloside IV | Review | 51 | 2017 | Fundamental & Clinical Pharmacology | ^3^ |
| 4 | Review of the Botanical Characteristics, Phytochemistry, and Pharmacology of Astragalus membranaceus (Huangqi) | Review | 43 | 2014 | Phytotherapy Research | ^4^ |
| 5 | A Review of the Pharmacological Action of Astragalus Polysaccharide | Review | 41 | 2020 | Frontiers in Pharmacology | ^5^ |
| 6 | Astragalus polysaccharides exerts immunomodulatory effects via TLR4-mediated MyD88-dependent signaling pathway in vitro and in vivo | Article | 36 | 2017 | Scientific Reports | ^6^ |
| 7 | Structural features and biological activities of the polysaccharides from Astragalus membranaceus | Review | 35 | 2014 | International Journal of Biological Macromolecules | ^7^ |
| 8 | The Antioxidant Effects of Radix Astragali (Astragalus membranaceus and Related Species) in Protecting Tissues from Injury and Disease | Review | 30 | 2016 | Current Drug Targets | ^8^ |
| 9 | Global cancer statistics 2018: GLOBOCAN estimates of incidence and mortality worldwide for 36 cancers in 185 countries | Article | 30 | 2018 | CA: A Cancer Journal for Clinicians | ^9^ |
| 10 | Astragali Radix (Huangqi): A promising edible immunomodulatory herbal medicine | Review | 28 | 2018 | Journal of Ethnopharmacology | ^10^ |
| 10 | Astragaloside IV derived from Astragalus membranaceus: A research review on the pharmacological effects | Review | 28 | 2018 | Advances in Pharmacology | ^11^ |

**Reference**

1. Auyeung KK, Han QB, Ko JK. Astragalus membranaceus: A Review of its Protection Against Inflammation and Gastrointestinal Cancers. *Am J Chin Med*. 2016;44(1):1-22. doi:10.1142/S0192415X16500014

2. Liu P, Zhao H, Luo Y. Anti-Aging Implications of Astragalus Membranaceus (Huangqi): A Well-Known Chinese Tonic. *Aging Dis*. Dec 2017;8(6):868-886. doi:10.14336/AD.2017.0816

3. Li L, Hou X, Xu R, Liu C, Tu M. Research review on the pharmacological effects of astragaloside IV. *Fundam Clin Pharmacol*. Feb 2017;31(1):17-36. doi:10.1111/fcp.12232

4. Fu J, Wang Z, Huang L, et al. Review of the botanical characteristics, phytochemistry, and pharmacology of Astragalus membranaceus (Huangqi). *Phytother Res*. Sep 2014;28(9):1275-83. doi:10.1002/ptr.5188

5. Zheng Y, Ren W, Zhang L, Zhang Y, Liu D, Liu Y. A Review of the Pharmacological Action of Astragalus Polysaccharide. *Front Pharmacol*. 2020;11:349. doi:10.3389/fphar.2020.00349

6. Zhou L, Liu Z, Wang Z, et al. Astragalus polysaccharides exerts immunomodulatory effects via TLR4-mediated MyD88-dependent signaling pathway in vitro and in vivo. *Sci Rep*. Mar 17 2017;7:44822. doi:10.1038/srep44822

7. Jin M, Zhao K, Huang Q, Shang P. Structural features and biological activities of the polysaccharides from Astragalus membranaceus. *Int J Biol Macromol*. Mar 2014;64:257-66. doi:10.1016/j.ijbiomac.2013.12.002

8. Shahzad M, Shabbir A, Wojcikowski K, Wohlmuth H, Gobe GC. The Antioxidant Effects of Radix Astragali (Astragalus membranaceus and Related Species) in Protecting Tissues from Injury and Disease. *Current drug targets*. 2016;17(12):1331-40. doi:10.2174/1389450116666150907104742

9. Bray F, Ferlay J, Soerjomataram I, Siegel RL, Torre LA, Jemal A. Global cancer statistics 2018: GLOBOCAN estimates of incidence and mortality worldwide for 36 cancers in 185 countries. *CA Cancer J Clin*. Nov 2018;68(6):394-424. doi:10.3322/caac.21492

10. Chen Z, Liu L, Gao C, et al. Astragali Radix (Huangqi): A promising edible immunomodulatory herbal medicine. *J Ethnopharmacol*. Aug 10 2020;258:112895. doi:10.1016/j.jep.2020.112895

11. Zhang J, Wu C, Gao L, Du G, Qin X. Astragaloside IV derived from Astragalus membranaceus: A research review on the pharmacological effects. *Adv Pharmacol*. 2020;87:89-112. doi:10.1016/bs.apha.2019.08.002
