# Supplementary material for: Research state of the herbal medicine Huangqi (Radix Astragali): A global and bibliometric study
Source: Medicine (Baltimore). 2024 Feb 23;103(8):e37277. doi: 10.1097/MD.0000000000037277 (PMC11309597; doi:10.1097/MD.0000000000037277)
Supplement: Supplementary file 6 [file medi-103-e37277-s006.docx]

**Table S6. Top 10 Co-cited references.**

| Rank | Title | Type | Co-Citated times | Year | Journal | Reference |
| --- | --- | --- | --- | --- | --- | --- |
| 1 | Review of the Botanical Characteristics, Phytochemistry, and Pharmacology of Astragalus membranaceus (Huangqi) | Review | 109 | 2014 | Phytotherapy Research | ^1^ |
| 2 | Chemical analysis of Radix Astragali (Huangqi) in China: A comparison with its adulterants and seasonal variations | Article | 85 | 2002 | Journal of Agricultural and Food Chemistry | ^2^ |
| 3 | A study on the immune receptors for polysaccharides from the roots of Astragalus membranaceus, a Chinese medicinal herb | Article | 80 | 2004 | Biochemical and Biophysical Research Communications | ^3^ |
| 4 | Astragalus membranaceus: A Review of its Protection Against Inflammation and Gastrointestinal Cancers | Review | 71 | 2016 | The American Journal of Chinese Medicine | ^4^ |
| 5 | TCMSP: a database of systems pharmacology for drug discovery from herbal medicines | Article (Database) | 66 | 2014 | Journal of Cheminformatics | ^5^ |
| 6 | Structural features and biological activities of the polysaccharides from Astragalus membranaceus | Review | 62 | 2014 | International Journal of Biological Macromolecules | ^6^ |
| 7 | In vitro and in vivo anti-tumor effects of Astragalus membranaceus | Article | 60 | 2007 | Cancer Letters | ^7^ |
| 8 | Antiinflammatory activity of astragaloside IV is mediated by inhibition of NF-kappa B activation and adhesion molecule expression | Article | 54 | 2003 | Thrombosis and Haemostasis | ^8^ |
| 9 | Astragaloside IV protects against ischemia brain injury in a murine model of transient focal ischemia | Article | 52 | 2004 | Neuroscience Letters | ^9^ |
| 10 | Pharmacological effects of Astragaloside IV: a literature review | Review | 52 | 2013 | Journal of Traditional Chinese Medicine | ^10^ |

**Reference**

1. Fu J, Wang Z, Huang L, et al. Review of the botanical characteristics, phytochemistry, and pharmacology of Astragalus membranaceus (Huangqi). *Phytother Res*. Sep 2014;28(9):1275-83. doi:10.1002/ptr.5188

2. Ma XQ, Shi Q, Duan JA, Dong TT, Tsim KW. Chemical analysis of Radix Astragali (Huangqi) in China: a comparison with its adulterants and seasonal variations. *J Agric Food Chem*. Aug 14 2002;50(17):4861-6. doi:10.1021/jf0202279

3. Shao BM, Xu W, Dai H, Tu P, Li Z, Gao XM. A study on the immune receptors for polysaccharides from the roots of Astragalus membranaceus, a Chinese medicinal herb. *Biochem Biophys Res Commun*. Aug 6 2004;320(4):1103-11. doi:10.1016/j.bbrc.2004.06.065

4. Auyeung KK, Han QB, Ko JK. Astragalus membranaceus: A Review of its Protection Against Inflammation and Gastrointestinal Cancers. *Am J Chin Med*. 2016;44(1):1-22. doi:10.1142/S0192415X16500014

5. Ru J, Li P, Wang J, et al. TCMSP: a database of systems pharmacology for drug discovery from herbal medicines. *J Cheminform*. 2014;6:13. doi:10.1186/1758-2946-6-13

6. Jin M, Zhao K, Huang Q, Shang P. Structural features and biological activities of the polysaccharides from Astragalus membranaceus. *Int J Biol Macromol*. Mar 2014;64:257-66. doi:10.1016/j.ijbiomac.2013.12.002

7. Cho WC, Leung KN. In vitro and in vivo anti-tumor effects of Astragalus membranaceus. *Cancer Lett*. Jul 8 2007;252(1):43-54. doi:10.1016/j.canlet.2006.12.001

8. Zhang WJ, Hufnagl P, Binder BR, Wojta J. Antiinflammatory activity of astragaloside IV is mediated by inhibition of NF-kappaB activation and adhesion molecule expression. *Thromb Haemost*. Nov 2003;90(5):904-14. doi:10.1160/TH03-03-0136

9. Luo Y, Qin Z, Hong Z, et al. Astragaloside IV protects against ischemic brain injury in a murine model of transient focal ischemia. *Neurosci Lett*. Jun 17 2004;363(3):218-23. doi:10.1016/j.neulet.2004.03.036

10. Ren S, Zhang H, Mu Y, Sun M, Liu P. Pharmacological effects of Astragaloside IV: a literature review. *J Tradit Chin Med*. Jun 2013;33(3):413-6. doi:10.1016/s0254-6272(13)60189-2
