# Supplementary material for: Research state of the herbal medicine Huangqi (Radix Astragali): A global and bibliometric study
Source: Medicine (Baltimore). 2024 Feb 23;103(8):e37277. doi: 10.1097/MD.0000000000037277 (PMC11309597; doi:10.1097/MD.0000000000037277)
Supplement: Supplementary file 7 [file medi-103-e37277-s007.docx]

**Table S7. Top 10 keywords.**

| Rank | Keyword | Frenquency |
| --- | --- | --- |
| 1 | Expression | 322 |
| 2 | Apoptosis | 261 |
| 3 | Oxidative stress | 217 |
| 4 | Cells | 203 |
| 5 | Inflammation | 198 |
| 6 | Astragaloside iv | 194 |
| 7 | In-vitro | 181 |
| 8 | Activation | 179 |
| 9 | Astragalus membranaceus | 141 |
| 10 | Traditional Chinese medicine | 133 |
